# Therapeutic Effect of Subunit Vaccine AEC/BC02 on *Mycobacterium tuberculosis* Post-Chemotherapy Relapse Using a Latent Infection Murine Model

**DOI:** 10.3390/vaccines10050825

**Published:** 2022-05-23

**Authors:** Jinbiao Lu, Xiaonan Guo, Chunhua Wang, Weixin Du, Xiaobing Shen, Cheng Su, Yongge Wu, Miao Xu

**Affiliations:** 1 National Engineering Laboratory for AIDS Vaccine, School of Life Sciences, Jilin University, Changchun 130012, China; lujinbiao@nifdc.org.cn (J.L.); xnguojlu@163.com (X.G.); 2 Division of Tuberculosis Vaccine and Allergen Products, Institute of Biological Product Control, National Institutes for Food and Drug Control, Beijing 102629, China; wchdye@126.com (C.W.); duwxin@126.com (W.D.); shengt99@163.com (X.S.); jhzz1225@hotmail.com (C.S.)

**Keywords:** tuberculosis, AEC/BC02 vaccine, latent tuberculosis infection, immunotherapy, relapse

## Abstract

Tuberculosis (TB), caused by the human pathogen *Mycobacterium tuberculosis* (*Mtb*), is an infectious disease that presents a major threat to human health. Bacillus Calmette-Guérin (BCG), the only licensed TB vaccine, is ineffective against latent TB infection, necessitating the development of further TB drugs or therapeutic vaccines. Herein, we evaluated the therapeutic effect of a novel subunit vaccine AEC/BC02 after chemotherapy in a spontaneous *Mtb* relapse model. Immunotherapy followed 4 weeks of treatment with isoniazid and rifapentine, and bacterial loads in organs, pathological changes, and adaptive immune characteristics were investigated. The results showed slowly increased bacterial loads in the spleen and lungs of mice inoculated with AEC/BC02 with significantly lower loads than those of the control groups. Pathological scores for the liver, spleen, and lungs decreased accordingly. Moreover, AEC/BC02 induced antigen-specific IFN-γ-secreting or IL-2-secreting cellular immune responses, which decreased with the number of immunizations and times. Obvious Ag85b- and EC-specific IgG were observed in mice following the treatment with AEC/BC02, indicating a significant Th1-biased response. Taken together, these data suggest that AEC/BC02 immunotherapy post-chemotherapy may shorten future TB treatment.

## 1. Introduction

Tuberculosis is a chronic infectious disease caused by *Mycobacterium tuberculosis* that is transmitted through the respiratory tract and remains one of the top 10 causes of mortality worldwide [1]. The World Health Organization has reported that the incidence of TB in 2019 was 10 million, including 1.2 million deaths among HIV-negative individuals and an additional 208,000 deaths among HIV-positive individuals [2]. China currently accounts for 8.4% of global TB patients, ranking third in the world. Particularly in light of the disruption to medical services caused by the coronavirus disease-2019 (COVID-19) pandemic, it may also have contributed to the neglect of known diseases such as tuberculosis, the knowledge of which was already less than ideal [3]. Further, it is also estimated that a quarter of the population in the world have latent tuberculosis infection (LTBI), and most cases of tuberculosis resulted from reactivation of LTBI [4]. Therefore, the prevention and control of LTBI has major implications for the approach to tuberculosis elimination [5].

Currently, Bacillus Calmette-Guérin (BCG) is the only available vaccine to prevent life-threatening TB disease in infants and children [6,7]. However, it has demonstrated limited and variable effectiveness (0% to 80%) in preventing pulmonary TB and in curbing the transmission of *Mtb* in adults and adolescents [8,9,10]. In addition, BCG is ineffective in containing reactivation of latent TB infection (LTBI) [11]. Chemotherapy of TB has a cure rate of approximately 85%, with shortcomings such as long treatment cycles and side effects [12,13]. Currently, immunotherapy as an adjunct to chemotherapy has been used in many experimental models to eliminate proliferation of latent infection *Mtb* by non-specific or antigen-specific immunological agents [14]. Relapse of *Mtb* in the lungs can be reduced with an immunotherapy regimen consisting of recombinant IFN-γ + IgA + anti-IL-4 or muramyl dipeptide followed by short-term (incomplete) chemotherapy [14,15]. Studies have found that vaccines can effectively reduce the post-chemotherapy relapse of *Mtb*-infected mice when using hsp65 or Ag85A DNA vaccine, or fragmented *Mtb* cells detoxified with liposomed (RUTI) [12,16,17,18]. On the basis of the efficacy of vaccines after chemotherapy, new therapeutic vaccines are a potential preventive or therapeutic method, as an adjunct to chemotherapy.

It is reported that Ag85b, ESAT6, and CFP10 are promising vaccine candidate molecules given that they are strongly recognized by T-cell antigens in the first phase of infection [19,20]. Previous studies demonstrated that Ag85b immunization can provide protection against TB infection, which is associated with increased IFN-γ production [21]. ESAT6 induces a strong cellular immune response that may provide protection in individuals with prior TB exposure [22,23], and CFP10 activates CD8^+^ T cells in vivo, which have persistent cytolytic activity after *Mtb* infection [24]. We have constructed a candidate TB vaccine, designated AEC/BC02, using the Ag85b antigen and ESAT6-CFP10 (EC) fusion protein, along with the new adjuvant BC02 from the BCG-derived unmethylated cytosine–phosphate–guanine (CpG) DNA fragment and aluminum salt. It has been demonstrated that BC02 was safe and increased both antigen-specific IL-12 and IFN-γ levels [25], and also induced innate immunity [26]. Interestingly, we found that AEC/BC02 cannot provide effective protection as a pre-exposure prophylaxis vaccine, possibly because the recombinant protein vaccine contained only a few antigens, which was not sufficient to elicit the equal protective immune effect to BCG, but AEC/BC02 can be used as an immunotherapeutic against progressive disease in a guinea pig LTBI model after chemotherapy [27]. However, existing studies are limited to the analysis of a single immunological index induced by AEC/BC02, while other immune effectors induced by the vaccine, the effects on the humoral immune response, and the immune response induced in cases of *Mtb* latent infection remain unclear. Therefore, in this study, we investigated the therapeutic effect and immunological properties in terms of humoral and cellular immunity of the candidate AEC/BC02 vaccine of post-chemotherapy against latent *Mtb* infection in a mouse model.

## 2. Materials and Methods

### 2.1. Bacterial Strains and Culture Conditions

The *Mtb* strain (CMCC 95052) used in this study was grown on Lowenstein Jensen medium at 37 °C for approximately 4 weeks. The turbidity of the bacterial suspensions was measured using a McNamara turbidimetric tube assay, and then bacteria were diluted to 1 mg/mL. The samples were stored at −70 °C in aliquots.

### 2.2. Mice

Specific-pathogen-free C57BL/6 mice (females, 6–8-weeks-old) were purchased from the Institute for Laboratory Animal Resources, National Institutes for Food and Drug Control (NIFDC). Mice were housed under barrier conditions at an Animal Biosafety Level III laboratory. All animals used in this study were treated according to the standards of animal welfare and procedures were reviewed by the Animal Care and Welfare Committee of the NIFDC.

### 2.3. Vaccine Preparation

To generate the recombinant tuberculosis vaccine AEC/BC02, Ag85b and fusion protein ESAT6-CFP10 were combined with Bacillus Calmette-Guérin CpG and the aluminum (InvivoGen, Cayla, France) adjuvant system. The *Mtb* antigen proteins and CpG were provided by Anhui Zhifei Longcom Biologic Pharmacy Company (Hefei, China).

One dose of AEC/BC02 vaccine comprised 10 μg of Ag85b and 10 μg of ESAT6-CFP10 (EC) in 0.2 mL of adjuvant BC02. Per mouse, the BC02 contained 0.2 mg of aluminum hydroxide and 75 μg of CpG in phosphate-buffered saline (PBS).

### 2.4. Study Design

**Figure 1:**
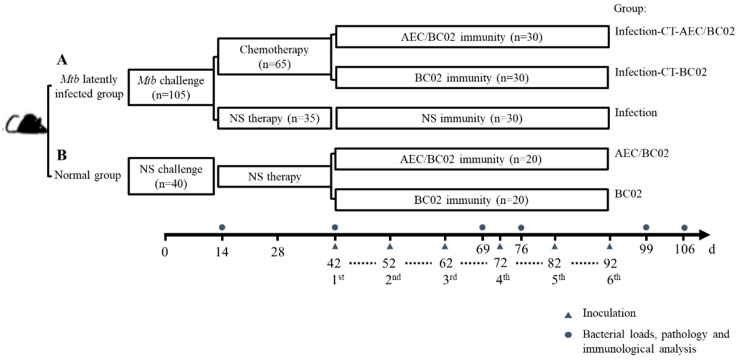
Immunotherapy schedule for evaluation of a potential vaccine candidate in a *Mtb* latently infected mouse model. Mice were randomly divided into groups A and B. (**A**) For *Mtb* latently infected group: mice were intraperitoneally challenged with 5 × 10^5^ CFU of *Mtb* (t = 0 day), followed by chemotherapeutic treatment twice a week for 4 weeks (2 weeks after challenge) or NS as an infection control. (**B**) Normal group: mice were injected and treated with NS on days 0 and 14. Except for the infection control group of group A that was not immunized, the other mice were separately inoculated with one dose of AEC/BC02 and BC02 six times at 10-day intervals. Mice in all groups were euthanized at 14, 42, 69, 76, 99, or 106 days for immunological, bacterial load, and pathological analysis.

One hundred and forty-five mice were randomly allocated into group A (*Mtb* latently infected group, *n* = 105) and group B (normal control group, *n* = 40). On day 0, mice in group A (Figure 1A) were intraperitoneally challenged with 0.5 mL of *Mtb* (5 × 10^5^ CFU). On day 14, mice were administered chemotherapy (CT) or normal saline (NS) therapy twice a week for four weeks by oral gavage. The administration volume for each mouse was 0.2 mL of isoniazid and rifapentine suspension, containing 0.5 mg and 0.2 mg, respectively. Mice in group B (Figure 1B) were injected intraperitoneally and treated orally with an equal volume of NS to that of group A. On day 42, mice subjected to the infection-CT-AEC/BC02 (*n* = 30), AEC/BC02 (*n* = 20) schedule, were administered intramuscularly (i.m.) with a dose of AEC/BC02 vaccine; mice subjected to the infection-CT-BC02 (*n* = 30), BC02 (*n* = 20) schedule, were administered i.m. with a dose of BC02; and mice subjected to the infection (*n* = 30) schedule were administered i.m. with 0.2 mL of NS. All mice were inoculated six times at 10-day intervals with a dosage volume of 0.2 mL. Finally, all animals were euthanized at pre-treatment (day 14), pre-immune (day 42), and 1 and 2 weeks after immunization 3 (day 66 and day 76) and 6 (day 99 and day 106), and were tested for immunogenicity, organ bacterial loads, and pathological analysis.

### 2.5. Determination of Bacterial Loads and Pathology

After the mice had been euthanized, the liver, spleen, and lungs were excised. Half of the harvested spleens and lungs were diluted with 0.5 mL of ground homogenate and 4.5 mL of normal saline and were plated on modified Lowenstein Jenson medium. The CFUs were determined after 4 weeks of incubation at 37 °C.

The other half of the lungs, spleens, and livers were examined for pathology. The tissues were embedded in paraffin blocks, sectioned at a 5 μm thickness, and stained with a hematoxylin and eosin (H&E) staining solution. The sections were then observed for lesions under an optical microscope. Pathological scores for the livers, spleens, and lungs were evaluated separately. The pathological changes of granuloma, edema, monocyte infiltration, and macrophage aggregation were semi-quantitatively scored. No lesions were scored as 0 points, very mild as 1 point, mild as 2 points, moderate as 3 points, and severe as 4 points. All pathological results in this experiment were scored by the same professional pathologist for blind analysis.

### 2.6. IFN-γ/IL-2 Enzyme-Linked Immunospot (ELISPOT) Assay

To quantify the number of cells secreting antigen-specific IFN-γ/IL-2 in the spleen, an ELISPOT assay was performed according to the manufacturer’s instructions (Mabtech AB, Nacka Strand, Sweden). In brief, freshly harvested splenocytes (2.5 × 10^5^/well) at 1 and 2 weeks after the third and sixth immunizations were stimulated with the synthesized Ag85b, ESAT6, and CFP10 peptide pools (15 amino acids, overlapping 10 amino acids, 2 μg/mL); concanavalin A (positive control, 1 μg/mL); or medium alone (negative control) for 48 h in a 37 humidified incubator with 5% CO_2_. At the end of the culture period, plates were washed five times with PBS (200 μL/well) and incubated for 2 h with the related biotinylated detection antibody. Streptavidin–horseradish peroxidase was then added for 1 h, followed by the substrate solution. The ELISPOT plates were incubated in the dark at room temperature until spots were visible, and dried after the reaction was terminated by washing with deionized water. The number of spot-forming cells (SFCs) was counted by an automated ELISPOT reader (Cellular Technology Ltd., Cleveland, OH, USA). The negative control was subtracted from the antigen-specific response at each time point, and the results were reported as the number of SFCs per 2.5 × 10^5^ splenocytes.

### 2.7. Determination of Antibody Titers

Serum samples were collected 1 and 2 weeks after the third and sixth immunizations to evaluate antibody levels. Antigen-specific IgG, IgG1, and IgG2a levels in serum were measured via an enzyme-linked immunosorbent assay (ELISA). The 96-well plates were coated with 0.1 μg of Ag85b or EC proteins per well and incubated overnight at 4 °C, then blocked with PBS containing 1% BSA at 37 °C for 2 h and washed with 0.05% Tween-20 in PBS. Sera were serially diluted twofold and incubated at 37 °C for 1 h. Then, the plates were washed, and the bound antibodies were reacted with HRP-conjugated goat anti-mouse IgG (1:5000) (Abcam), IgG1 (1:5000) (Alpha Diagnostic International), and IgG2a (1:10,000) (Alpha Diagnostic International) at 37 °C for 1 h, followed by the addition of 3,3′,5,5′-tetramethylbenzidine (TMB) substrate solution. The reaction was stopped by the addition of 2 M H_2_SO_4_, and the optical density (OD) of the samples was measured at 450 nm, with 620 nm as the reference wavelength. The endpoint titers were calculated as the reciprocal of the last dilution reaching a cut-off value set to 2.1-fold above the mean optical density of a negative control.

### 2.8. Statistical Analysis

Statistical analyses were performed using GraphPad Prism 8.0 (GraphPad Software), and the statistical significance of the difference between two groups was performed using the *t*-test or Mann–Whitney U test, with one-way ANOVA or Kruskal–Wallis test for the analysis of differences between multiple groups. The results are expressed as mean ± SEM. Significant differences were established if *p* < 0.05. */^#^*p* < 0.05, or **/^##^*p* < 0.01.

## 3. Results

### 3.1. Therapeutic Effect of the AEC/BC02 Vaccine on the Bacterial Loads in Latently-Infected Mice

**Figure 2:**
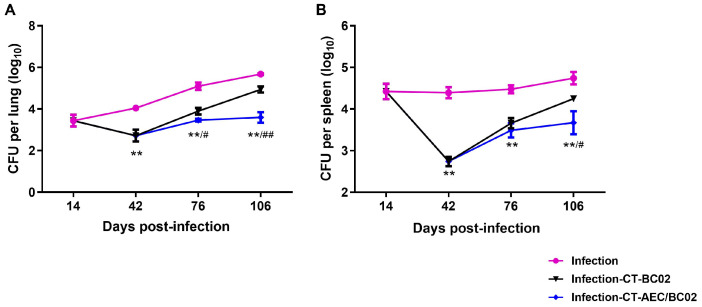
Evaluation of the immunotherapeutic effects of AEC/BC02 vaccine after chemotherapy in the *Mtb* latent infection mouse model. Bacterial loads in the lungs (**A**) and spleens (**B**) were measured in mice. Data are shown as the mean ± SEM. ** The infection-CT-AEC/BC02 group was compared with the infection group. ^#^/^##^ The infection-CT-AEC/BC02 group was compared with the infection-CT-BC02 group. Statistical significance was set at ^#^ *p* < 0.05 or **/^##^ *p* < 0.01 (*n* = 5).

To evaluate the therapeutic effect of AEC/BC02 vaccine after chemotherapy on mice with *Mtb* latent infection, the bacterial loads in the lungs and spleens of mice on different time points were analyzed. As shown in Figure 2, 2 weeks after *Mtb* infection of mice, the bacterial counts in the lungs and spleens were 3.44 ± 0.28 log_10_ CFU and 4.42 ± 0.18 log_10_ CFU, respectively. The counts of bacteria in the lungs and spleens of the infected group without chemotherapy or immunotherapy, increased gradually with time, while the number of bacteria in the infected mice after 4 weeks of chemotherapy (day 42) declined significantly.

In contrast, the bacterial burden in the organs of each group gradually increased after chemotherapy, but this burden was lower in the infection-CT-AEC/BC02 group than in the infection-CT-BC02 group and differed significantly from the infection group (*p* < 0.01). Two weeks after the third dose of immunization (76 days), mice in the infection-CT-AEC/BC02 group had a significantly lower bacterial load in the lungs and lower bacterial load in the spleen compared with the infection-CT-BC02 group (0.43 ± 0.17 log_10_ CFU, *p* < 0.05 and 0.17 ± 0.13 log_10_ CFU reduction, respectively). Similar but lower reductions were observed in the lungs and spleen two weeks after the sixth immunization (106 days), with the bacterial load being significantly lower in the infection-CT-AEC/BC02 group than the infection-CT-BC02 group (1.34 ± 0.30 log_10_ CFU reduction, *p* < 0.01 and 0.58 ± 0.29 log_10_ CFU reduction, *p* < 0.05). These results indicated that AEC/BC02 immunotherapy can effectively inhibit the proliferation of *Mtb* in mice with latent *Mtb* infection and provide effective immune protection against *Mtb*.

### 3.2. Histological Changes and Lesion Scoring in Latently Infected Mice following the Treatment with AEC/BC02

**Figure 3:**
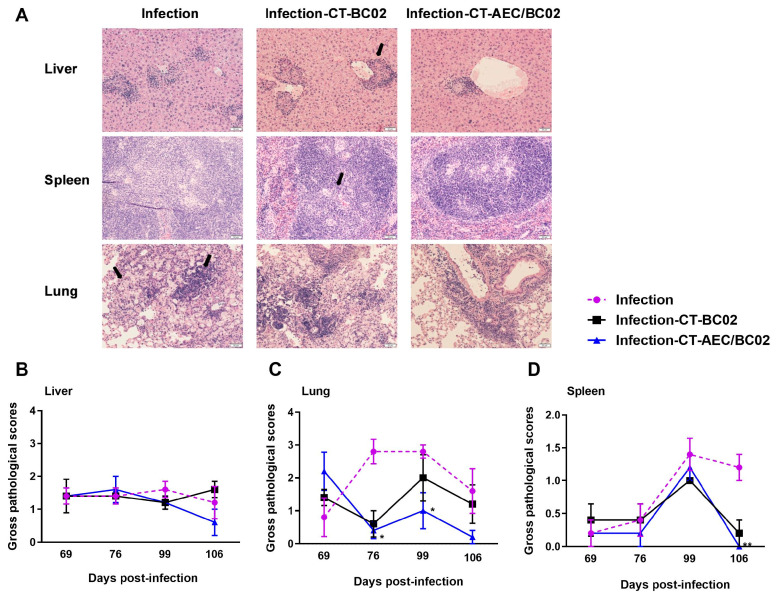
Evaluation of the immunotherapeutic effects of AEC/BC02 vaccine on tissue pathology in the livers, lungs, and spleens in *Mtb* latently infected mice. (**A**) Pathological changes in the livers, lungs, and spleens of mice. Black arrows indicate granulomatous lesions of the livers and spleens, as well as the infiltration of monocytes and the accumulation of macrophages in the lungs. Pathological scores are shown for the livers (**B**), lungs (**C**), and spleens (**D**) of mice. The infection-CT-AEC/BC02 group was compared with the infection group, and statistical significance was set at * *p* < 0.05 or ** *p* < 0.01 (*n* = 5).

Histological findings revealed that the livers and spleens of mice mainly developed granulomatous lesions, and the lungs mainly showed pathological changes involving monocyte infiltration, macrophage aggregation, and edema (Figure 3A). Semi-quantitative scoring was performed for the above lesions, and the detailed results are described in Figure 3B–D. From 69 days to 106 days after infection, the pathological scores for the livers, lungs, and spleens in the infection-CT-AEC/BC02 group gradually decreased below those of the infection and infection-CT-BC02 groups. Statistically significant differences were found in the pathological scores for lungs between the infection-CT-AEC/BC02 and infection groups at 76 and 99 days after infection (*p* < 0.05). Furthermore, the pathological scores for spleens of the infection-CT-AEC/BC02 and infection groups also showed a significant difference on day 106 after infection (*p* < 0.01). Taken together, these data indicated that the AEC/BC02 vaccine could reduce tissue lesions and thus provided a degree of protection in mice latently infected with *Mtb*.

### 3.3. Cellular Immune Response in Mouse Models

**Figure 4:**
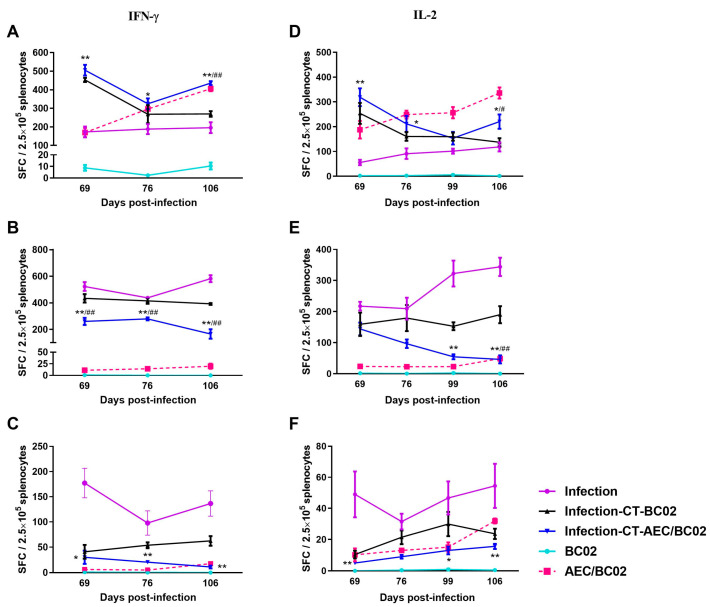
The frequency of spot-forming cells expressing IFN-γ or IL-2 was determined by ELISPOT. The magnitude of the IFN-γ-secretion response produced by stimulation of splenic lymphocytes in normal mice and *Mtb* latently infected mice with Ag85b (**A**), ESAT6 (**B**), and CFP10 (**C**) peptides. The secretion of IL-2 cells stimulated by Ag85b (**D**), ESAT6 (**E**), and CFP10 (**F**) peptides was detected in the splenocytes of the two mouse models. */** The infection-CT-AEC/BC02 group was compared with the infection group. ^#^/^##^ The infection-CT-AEC/BC02 group was compared with the infection-CT-BC02 group. Statistical significance was set at */^#^ *p* < 0.05 or **/^##^ *p* < 0.01 (*n* = 5).

To further investigate the cell-mediated response induced by AEC/BC02, the number of antigen-specific IFN-γ/IL-2-secreting cells was determined in the splenocytes of mice re-stimulated with the Ag85b/ESAT6/CFP-10 peptide by ELISPOT. As shown in Figure 4, in the normal mouse model, specific IFN-γ/IL-2-secreting cells were almost undetectable in the BC02 group, while the number of specific IFN-γ/IL-2 SFCs was significantly higher in the AEC/BC02 group and increased with the increase in immunization dose and time.

In the *Mtb* latently infected mouse model, the number of Ag85b/ESAT6-specific IFN-γ/IL-2 cells and the number of CFP10-specific IFN-γ cells decreased with increased immunization dose and time, while the number of CFP10-specific IL-2 cells increased in the infection-CT-AEC/BC02 group. Furthermore, the number of Ag85b-specific IFN-γ/IL-2 cells was higher than that in the infection-CT-BC02 and infection groups, and the number of ESAT6/CFP10-specific IFN-γ/IL-2 cells showed the opposite trend. In addition, the Ag85b/ESAT6/CFP10-specific IFN-γ cell immune response and the ESAT6-specific IL-2 cell immune response induced by AEC/BC02 in the *Mtb* latent infection mice were both higher than those in normal mice, while Ag85b/CFP10-specific IL-2 cell immunity was lower than that in normal mice. Taken together, a long-term specific cellular immune response could be induced in all groups of *Mtb* latently infected mice and the AEC/BC02 group of normal mice.

### 3.4. Humoral Immune Response in Mouse Models

**Figure 5:**
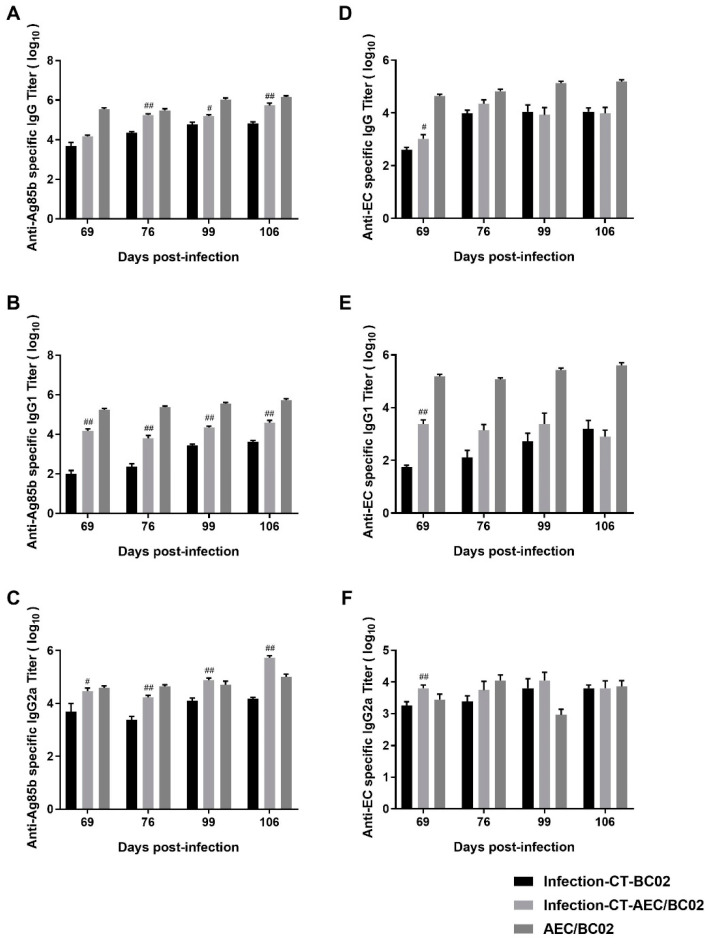
Characteristics of the humoral immune responses in *Mtb* latently infected and normal mice immunized with AEC/BC02. Ag85b-specific IgG (**A**), IgG1 (**B**), and IgG2a (**C**) in the infection-CT-BC02, infection-CT-AEC/BC02, and AEC/BC02 immunized mice were determined by ELISAs. EC-specific IgG (**D**), IgG1 (**E**), and IgG2a (**F**) in the infection-CT-BC02, infection-CT-AEC/BC02, and AEC/BC02 immunized mice were determined by ELISAs. Data from the infection-CT-AEC/BC02 group were compared with the infection-CT-BC02 group, and statistical significance was set at ^#^ *p* < 0.05, ^##^ *p* < 0.01 (*n* = 5).

To evaluate the level of humoral immunity induced by the AEC/BC02 vaccine, antigen-specific IgG, IgG1, and IgG2a antibody levels were determined in the serum of test mice 1 and 2 weeks after the third and sixth immunizations with latently-infected *Mtb* and normal mice. As shown in Figure 5, the Ag85b- and EC-specific IgG antibodies and subtypes were detectable in the sera of all groups, and high antibody titers were only detected in the infection-CT-AEC/BC02, infection-CT-BC02, and AEC/BC02 groups, and no positive antibody was detected in the other groups. For specific anti-Ag85b antibodies (Figure 5A–C), the antibody titers of the three groups increased along with the increase in immunization dose and time, and the antibody levels of IgG and IgG1 were highest in the AEC/BC02 group, followed by the infection-CT-AEC/BC02 group, and lastly the infection-CT-BC02 group. However, the titer of IgG2a increased in the infection-CT-AEC/BC02 group, eventually exceeding that of the AEC/BC02 group over time. With the exception of the anti-Ag85b IgG titer at 69 days, the IgG titers in the vaccine groups were significantly higher than in the adjuvant group (*p* < 0.05 or *p* < 0.01). For specific anti-EC antibodies (Figure 5D–F), the titers and antibody intensities were similar to Ag85b, except that the infection-CT-AEC/BC02 group antibodies showed a slight downward trend over time. The infection-CT-AEC/BC02 group showed a slight increase in specific anti-EC antibodies compared with the infection-CT-BC02 group, but no statistical significance was detected, with the exception of the anti-EC antibody titers at 69 d. In conclusion, the AEC/BC02 vaccine could induce a high level of humoral immune response in normal mice and significantly enhance antibody levels in *Mtb* latently infected mice compared with the adjuvant group.

**Figure 6:**
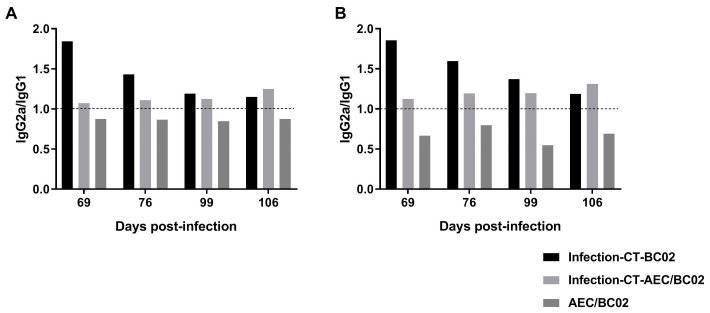
The immunological types of *Mtb* latently infected and normal mice induced by AEC/BC02. (**A**) The ratio of Ag85b-specific IgG2a/IgG1. (**B**) The ratio of EC-specific IgG2a/IgG1. IgG2a/IgG1 > 1 indicates Th1-type bias, whereas IgG2a/IgG1 < 1 indicates Th2-type bias.

Furthermore, we analyzed the ratio of IgG2a/IgG1 in each group, as shown in Figure 6, and the immune response induced by AEC/BC02 in normal mice was biased towards Th2, while the immune response induced in *Mtb* latently infected mice was biased towards Th1. Furthermore, the Th1 immune response was increased with prolonged immunization time. BC02 could also induce a Th1-biased immune response in mice latently infected with *Mtb*, but this type of response gradually decreased with time.

## 4. Discussion

TB remains the most common cause of death from a single infectious pathogen [2], highlighting the urgent need for safer and more effective vaccines to control the spread of TB, especially because the commercially available vaccine BCG is ineffective in adults and LTBI. Due to the presence of latent *Mtb* in lesions, chemotherapy is less efficacious [28]. Previous studies have shown the AEC/BC02 vaccine to not be used as a pre-exposure preventive vaccine. However, the vaccine can inhibit *Mtb* infection in guinea pigs latently infected after chemotherapy, suggesting the potential of AEC/BC02 as an adjuvant therapy after chemotherapy [27]. Analyzing the immunological characteristics of this type of vaccine may help our understanding of the immune mechanism involved in *Mtb* infection and potential immunological indicators related to protective efficacy.

The intraperitoneal infection model has similar characteristics to low-dose aerosol infection, such as the establishment of stable CFU levels in organs after a period of challenge that does not change significantly over time [29]. Dhillon et al. reported that after 4 weeks of intraperitoneal challenge in mice, bacteria isolated from organs had some similarities to dormant *Mtb* obtained in vitro, suggesting the formation of a dormant (latent) bacterial population in this model [30]. In addition, intraperitoneal challenge also has the advantages of low levels of cross-contamination between animals and low experimental costs. Therefore, in this study, we used the less commonly used intraperitoneal challenge. The effect of AEC/BC02 immunotherapy was evaluated by organ load and pathological score. The results showed that the bacterial load of the spleen and lungs of the AEC/BC02 vaccine group was significantly decreased compared with the BC02 control group in a mouse model of latent infection after chemotherapy. Bacterial load was significantly lower than that of the infection control group, indicating that the immune response induced by AEC/BC02 may elicit a protective response to inhibit or delay the recurrence of *Mtb* in mice. Alternatively, the effectiveness of the vaccine may be related to the previous chemotherapy, on the basis of the observations of our previous study that immunization with the AEC/BC02 vaccine alone could not effectively reduce the number of bacteria in the organs [27]. This may be linked to the reduction of the immune response by chemotherapy, enabling the AEC/BC02 vaccine induced immune response to be effective [31]. AEC/BC02 vaccine may enhance previous immune responses or induce new immune responses against *Mtb* antigens. The increase in bacterial load in the chemotherapy + adjuvant group was higher than that in the vaccine group due to lack of sufficient immune response. Furthermore, the pathological scores for the liver, spleen, and lungs were also decreased in the AEC/BC02 vaccine group. It is worth noting that the pathological score of the BC02 group was also reduced, but higher than that of the vaccine group, which may have been related to the innate immune response induced by the BC02 adjuvant [26].

The cellular immune response is involved in protecting humans and animals from *Mtb* infection [32,33]. IFN-γ is an important effector that stimulates the production of reactive nitrogen intermediates and reactive oxygen intermediates, which further promote the maturation of phagocytes, thereby activating macrophages and clearing *Mtb*-infected cells [34,35,36]. IL-2 can not only maintain the survival of memory cells, but also plays an important role in the clonal proliferation and differentiation of T cells after antigen recognition, as well as promoting the activation of macrophages [37]. The antigen-specific IFN-γ and IL-2 secreting cells produced by the splenocytes of mice in each group were quantified by ELISPOT. We found that the production of Ag85b-specific IFN-γ/IL-2 in the vaccine group was upregulated compared with the control group and was negatively correlated with the proliferation of bacteria in the organs of latently infected mice. This result indicates that the Ag85b-specific Th1 response may be more important for protection from recurrence of *Mtb*. Interestingly, we also found that the number of ESAT6/CFP10-specific IFN-γ/IL-2 cells was greater in the infection group than in the BC02 adjuvant group, or the AEC/BC02 vaccine group. One possible hypothesis is the reduction of ESAT6/CFP10 expression by bacteria in response to chemotherapy-induced stress. Studies have shown that direct injection of Th1-polarized ESAT-6-specific CD4^+^ T cell clones cannot reduce bacterial loads in chemotherapeutic *Mtb*-infected mice [38]. The level of MHC-II associated ESAT-6 peptide on the surface of infected cells is reduced, thereby limiting the response of these lymphocytes. AEC/BC02 immunization may further reduce the expression of ESAT-6/CFP-10, resulting in downregulation of ESAT6/CFP10-specific IFN-γ/IL-2 cells. Ag85b, contrary to ESAT6/CFP10, may have enhanced expression during the *Mtb* chronic infection stage. It is of note that the immune response of IFN-γ/IL-2-secreting lymphocytes induced by AEC/BC02 in the latently infected mice decreased gradually with increased immunization dose and time. At present, there are limited reports evaluating the immunogenicity and immune efficacy of vaccines in infected animals. Alyahya et al. [39] reported that the cellular immune response and bacterial loads in tissues were decreased in mice following antibiotic treatment. In conclusion, the vaccine group showed similar changes in cellular immunology and organ bacterial loads to the antibiotic treatment group in the study by Alyahya and colleagues, suggesting that the AEC/BC02 vaccine provided effective anti-TB immune protection against latent *Mtb* infection in mice.

Normal and *Mtb* latently infected mice were immunized with AEC/BC02 or BC02, and serum samples were collected 1 and 2 weeks after the third and sixth immunizations to detect antibody levels. The results showed that AEC/BC02 vaccine could induce high titers of Ag85b- and EC-specific IgG, IgG1, and IgG2a antibodies with increased immunization doses and time. Surprisingly, we found high levels of antibodies in the infection-CT-BC02 group, but no antibodies were detected in the infection group. A possible explanation is that the long-term presence of high *Mtb* loads in infected mice may lead to rapid depletion and impaired function of B cells [40], so that antibody levels do not reach the limit of detection at the later stage of infection. However, the presence of chemotherapy in the infection-CT-BC02 group can reduce the bacterial load, possibly improve the in vivo environment, and restore the function of B cells. The presence of CpG in BC02 adjuvant may also play a role in the production of antibodies, and the specific mechanism remains to be explored. Some studies have reported that high levels of antibodies can prolong the survival of animals infected with *Mtb* [41,42], and can reduce the bacterial loads in infected tissues during the early stage of *Mtb* infection [43,44]. Therefore, it is speculated that specific antibodies induced by AEC/BC02 may be beneficial to the control of *Mtb* in latently infected animals. In addition, the levels of IgG and IgG1 antibodies induced in mice with latent *Mtb* infection were lower than those in normal mice, with the exception of IgG2a. Furthermore, the immune response induced by AEC/BC02 in *Mtb* latently infected mice showed a Th1 bias, indicating that the immune response in infected mice was mainly a cellular immune response. In addition, the antibody level was lower than that in normal mice, which may be related to the role of antibodies during the process of anti-*Mtb* infection. It is known that specific antibodies can recognize the surface antigen of *Mtb*, and their Fc segments can bind to the Fc receptors on the surface of killer cells, thereby mediating antibody-dependent cell-mediated cytotoxicity. Furthermore, the binding of *Mtb* to Fc receptors can guide the uptake of *Mtb* by macrophages and stimulate the activation of antigen-presenting cells, thus activating a series of subsequent specific immune reactions. It can also increase reactive oxygen intermediate products and promote the fusion of phagocytes and lysosomes [45,46,47,48]. Therefore, the antibody response may have a greater role in *Mtb* infection.

The limitation of this study is that only one route of infection with *Mtb* were used in the animal model. The candidate therapeutic vaccine AEC/BC02 should be further investigated in more animal models with other infection route in future.

In summary, this study revealed that AEC/BC02 immunotherapy could provide effective protection against *Mtb* latently infected mice after chemotherapy. It was confirmed that the vaccine induced a Th1-biased immune response in infected organisms, and this antigen-specific cellular immune response decreased with time. Our findings provided reference data for future research on the immunological characteristics of AEC/BC02 with respect to latent *Mtb* infection.

## Data Availability

The datasets generated for this study are available on request to the corresponding author.
